# Lauric Acid Is a Potent Biological Control Agent That Damages the Cell Membrane of *Phytophthora sojae*

**DOI:** 10.3389/fmicb.2021.666761

**Published:** 2021-08-05

**Authors:** Changhui Liang, Wenteng Gao, Ting Ge, Xinwei Tan, Jiayu Wang, Huaxin Liu, Yong Wang, Chao Han, Qian Xu, Qunqing Wang

**Affiliations:** ^1^Shandong Province Key Laboratory of Agricultural Microbiology, Department of Plant Pathology, College of Plant Protection, Shandong Agricultural University, Tai’an, China; ^2^Shimadzu (China) Co., Ltd., Beijing, China; ^3^State Key Laboratory of Crop Biology, Shandong Agricultural University, Tai’an, China

**Keywords:** lauric acid, leaf volatile compounds, *Phytophthora sojae*, cell membrane damage, biological control

## Abstract

Sustainable management of plant pathogens is becoming more challenging, and novel solutions are needed. Plant biologically active secondary metabolites are important sources of novel crop protection chemistry. Effective individual compounds of these natural products have the potential to be successful new agrochemicals. In this study, we identified lauric acid (LA) from soybean defense leaf volatiles. LA inhibited the growth of *Phytophthora sojae*, the causal agent of soybean root rot. It influenced mycelial development, sporangium formation, and zoospore generation and germination by damaging the *P. sojae* cell membrane. Additionally, we showed that LA and several of its derivatives, such as glycerol monolaurate (GML), had similar biological activities. Both LA and GML were safe to soybean plants when used at less than 0.3 g a.i./plant and could promote soybean growth, implying their potential as eco-friendly biological control agents.

## Introduction

Oomycetes are fungus-like eukaryotic organisms that belong to the class Saprolegniomycetidae of the kingdom Stramenopila (Yutin et al., 2008). They encompass notorious plant disease agents, including *Phytophthora*, *Pythium*, and *Albugo*, and a group of downy mildews (Tyler, 2001). Oomycetes have a negative impact on natural and farm ecosystems due to their strong pathogenicity and infectivity (Kamoun et al., 2015). In addition to the well-known potato late blight caused by *Phytophthora infestans*, which led to the 19th-century Irish famine, the persistence of sudden oak death caused by *Phytophthora ramorum* and grape downy mildew caused by *Plasmopara viticola* demonstrate that oomycete phytopathogens are a persistent threat to subsistence and commercial farming and destructive to native plants (Erwin and Ribeiro, 1996). Soybean (*Glycine max* L.) root rot caused by *Phytophthora sojae* is the leading cause of global soybean production loss (Tyler, 2007). Agrochemicals are largely used to control oomycete diseases, resulting in the emergence of resistant strains and resurgence events (Randall et al., 2014). The effective and sustainable control of oomycete-driven diseases requires the identification of novel pharmaceuticals and pesticides to drive the design of fungicides compatible with integrated pest management (IPM) approaches (Gessler et al., 2011).

Environmental-friendly botanical fungicides are valuable because of their higher efficiency, lower residue, and lower toxicity. Multiple interdisciplinary studies and abundant resources have been applied to find new and effective alternatives for the IPM of various oomycete species. Historically, traditional healers have used plants to prevent or cure infections, and active elements for disease control have been identified in various species. For example, artemisinin, present in sweet wormwood (*Artemisia annua*), a Chinese medicinal plant, is an effective antimalarial agent (Tu, 2011). Medium-chain fatty acids (MCFAs), including octanoic acid (C8), capric acid (C10), and lauric acid (LA) (C12), distilled from virgin coconut oil, inhibit various bacterial, fungal, and viral pathogens and have been widely used in human and veterinary medicine (Kabara et al., 1972; Bartolotta et al., 2001; Rouse et al., 2005; Yang et al., 2009, 2018; Shilling et al., 2013; Fortuoso et al., 2019). For example, capric acid kills *Candida albicans* quickly and effectively (Bergsson et al., 2001). LA is the most active MCFA at lower concentrations and after longer incubation times (Bergsson et al., 2001; Lalouckova et al., 2021). Additionally, LA has antimicrobial activity against both Gram-positive and Gram-negative pathogens, including *Staphylococcus aureus*, *Streptococcus mutans*, *Streptococcus pyogenes*, *Escherichia coli*, and *Helicobacter pylori* (Kabara et al., 1972; Rouse et al., 2005). In addition to their effect on human and animal pathogens, MCFAs also inhibit mycelial growth and spore germination of four plant pathogenic fungi: *Alternaria solani*, *Colletotrichum lagenarium*, *Fusarium oxysporum* f. sp. *Cucumerinum*, and *F. oxysporum* f. sp. *lycopersici* (Liu et al., 2008). These observations suggest that MCFAs may be useful for developing alternative IMP approaches for the control of phytopathogens.

Phytochemicals have a long history as sources of novel agrochemicals. Phytopathologists search for natural products (NPs) that can be developed into pesticides for the treatment of plant diseases. For example, poacic acid, which is commonly found in grass lignocellulosic hydrolyzates, inhibits the growth of *Sclerotinia sclerotiorum* and *A. solani* fungi and *P. sojae* oomycetes (Piotrowski et al., 2015). There is considerable evidence for direct protective effects of chemicals isolated from plant root or leaf exudates and leaf or fruit volatile organic compounds (VOCs) in various organisms. For example, ε-viniferin, isolated from *Vitis vinifera* canes, has antifungal activity against *P. viticola* and *Botrytis cinerea* (Schnee et al., 2013). Secomicromelin, 7-methoxy-8-(4′-methyl-3′-furanyl) coumarin, micromarin B, and isomicromelin, present in *Micromelum falcatum* fruits, inhibit *Pythium insidiosum* mycelial growth (Suthiwong et al., 2014). Cuminic acid, present in *Cuminum cyminum* L. seeds, inhibits *Phytophthora capsici* mycelial growth and zoospore germination (Wang et al., 2016). Gossypol, which is naturally present in cotton root tissues, has a strong inhibitory activity on the growth of various soil-borne oomycetes and fungi, including *Pythium irregulare*, *Pythium ultimum*, and *F. oxysporum* (Mellon et al., 2014). Several leaf VOCs are produced and emitted rapidly when plants respond to stresses, such as herbivore or mechanical damage or attack by necrotrophic fungi (Cowan, 1999; Scala et al., 2013; Matsui and Koeduka, 2016; Tanaka et al., 2018; Wang et al., 2020). These phytochemicals may contribute directly to plant defense by preventing pathogen invasion (Matsui, 2006; Kishimoto et al., 2008).

Here, we analyzed soybean leaf volatiles derived from compatible (disease-producing) and incompatible (successful plant defense) interactions with *P. sojae*, the causal agent of soybean root and stem rot disease. LA was identified by headspace solid-phase microextraction coupled with gas chromatography–mass spectrometry (HS-SPME-GC–MS) among the incompatible interaction-produced volatiles. Both LA and glycerol monolaurate (GML), a chemical compound formed from LA and glycerol, inhibited the mycelial growth of *P. sojae* in Petri dish assays. GML also disrupted or disintegrated the *P. sojae* plasma membrane, leading to shrunken mycelia and cytoplasmic electrolyte leakage. We provide a conclusion about the functions and the protective mechanisms of LA as a potential oomycete biological control agent.

## Materials and Methods

### Plant and *P. sojae* Cultivation

Soybean plants were cultivated in a growth chamber at 25℃, with a cycle of 16 h of high light intensity and 8 h of darkness. *P. sojae s*trains were grown on 10% V8 medium (10% V8 juice, 0.02% CaCO_3_, and 1.5% agar) at 25℃ in the dark. Hyphal plugs were cultured in V8 liquid medium for mycelial harvest. The mycelia were washed with sterile tap water three times and cultured in the darkness at 25℃ for 6 h for zoosporangium incubation and zoospore release. Finally, the concentration of the zoospore suspension was adjusted to 1 × 10^5^ mL.

### GC–MS Analysis

Soybean leaves inoculated with *P. sojae* were placed in a 20-mL headspace bottle. An AOC-6000 Multifunctional Autosampler (Shimadzu, Kyoto, Japan) was used for SPME injection, and a GCMS-TQ8040 NX (Shimadzu) was used for detection using the following standard SPME parameters: SPME fiber, FIB-C-WR-95/10; aging temperature, 240℃; aging time before extraction, 30 min; equilibration temperature, 40℃; equilibration time, 5 min; extraction time, 30 min; injection port temperature, 250℃; desorption time, 2 min; and aging time after extraction, 5 min. The GC–MS parameters used were as follows: column, inert cap pure-wax, 30 m × 0.25 mm × 0.25 m; oven program, 50℃(5 min) and 10℃/min 250℃(10 min); carrier gas pressure, 83.5 kPa; injection mode, split; split ratio, 5:1; ion-source temperature, 200℃; interface temperature, 250℃; detector voltage, tuning voltage +0.3 kV; and acquisition mode, MRM.

### Inhibitory Effects of LA and Its Derivatives on *P. sojae* Growth

Hyphal plugs with a 6 mm were cultured in V8 liquid medium containing different concentrations (0.5, 1, 1.5, 2, and 2.5 mM) of LA, its derivatives–GML, methyl laurate (MEL), and ethyl laurate (ETL)–or the same volume of sterile water as a control reference (CK). The inoculated medium was incubated in the darkness at 25℃ for 5 days. Then, the colony diameter was measured, and the mycelium status was observed under a light microscope.

### Effects of LA and Its Derivatives on *P. sojae* Zoosporangium Forming and Zoospores Release

Different concentrations of LA or its derivatives, GML, MEL, and ETL, were added into washed mycelia cultured in V8 liquid medium. After incubation at 25℃ for 6 h, the number of zoosporangia was determined under a light microscope.

Lauric acid and its derivatives were added into the same number of sporangium culture dishes without zoospore release. The zoospores were cultured at 25℃, and the number of zoospores was determined under a light microscope.

### Effects of LA and Its Derivatives on *P. sojae* (R2) Zoospore Germination

A 0.1-mL spore suspension was evenly coated on V8 medium containing different concentrations of LA or its derivatives, GML, MEL, and ETL. After incubation at 25℃ for 4 days, colony formation was analyzed visually.

### Damage of the *P. sojae* Cell Membrane by LA and Its Derivatives

Mycelia were cultured in V8 liquid medium for 3 days before different concentrations of LA, and its derivatives, GML, MEL, and ETL, were added to the medium. After 1–2 h of treatment, 1/10 of the total volume of propidium iodate (PI) was added to the culture. After 20 min of incubation, the mycelia were rinsed with PBS 2–3 times. The mycelium was observed under a fluorescence microscope.

The same mycelia were placed in liquid V8 medium containing different concentrations of LA or its derivatives, and the concentrations of DNA and protein, and the electrical conductivity, were measured every 2 h and plotted for analysis.

### Safety of LA and Its Derivatives on Soybean Plants

Different concentrations (0.5, 1, 1.5, 2, and 2.5 mM) of LA or its derivatives, GML, MEL, and ETL, were mixed with the same volume of soil. Sterile water was used as a blank control (CK), and each treatment was repeated three times. The growth and development of soybean seedlings were recorded 7 and 14 days after planting.

### Use of LA and Its Derivatives as Preplant Soil Fumigants

Different concentrations (0.5, 1, 1.5, 2, and 2.5 mM) of LA or its derivatives, GML, MEL, and ETL, were mixed with the same volume of soil. Sterile water was used as a blank control. The soil was covered with a plastic film; after 15 days, the plastic film was removed, and the soil was ventilated for 2 days. Soybeans were then planted, and their height was measured after 5 days.

## Results

### Lauric Acid Is Induced in Incompatible Soybean–*P. sojae* Interactions

Soybean is the main host of *P. sojae*. Therefore, soybean cultivars that contain resistance genes to *P. sojae* (*Rps*) are important resources in agricultural production. Resistance genes provide defense ability against pathogen varieties and induce the emergence of hypersensitive responses and the production of secondary metabolites.

We analyzed the leaf volatiles produced during incompatible soybean–*P. sojae* interactions. After inoculation with *P. sojae*, the main constituents of the volatiles produced from the leaves of the susceptible soybean cultivar Williams (without *Rps* genes) and the resistant cultivar Williams 82 (with the resistance gene *Rps1k*) were determined by GC–MS. The LA concentration in the leaf volatiles of Williams 82 was significantly higher than that of Williams (Supplementary Figure 1), suggesting that LA contributes to the defense response of soybean to *P. sojae*.

### Lauric Acid Is Toxic to *P. sojae*

**FIGURE 1:**
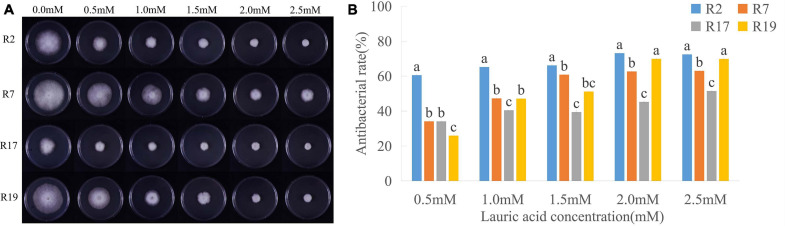
Inhibitory effects of lauric acid (LA) to various *P. sojae* species. **(A)** Four different *P. sojae* races (Race2, Race7, Race17, and, Race19) were cultured on V8 medium with different concentrations of LA for 5 days. The experiment was repeated three times with similar results. **(B)** The diameter of each colony was measured and statistical analyzed via software Statistical Product and Service Solutions (SPSS). Means with different letters are significantly different (*p* < 0.05).

We measured the radial growth diameters of different *P. sojae* races grown on V8 medium containing different concentrations of LA; the toxicity of LA to *P. sojae* was conspicuous (Figure 1 and Supplementary Table 1). After 5 days’ growing on V8 medium with 0.5 mM LA, the diameter of Race2 (strain P6497) hypha was 26.27 mm, and the bacteriostatic rate was 54.21%. Furthermore, the bacteriostatic rates of Race7 (strain P7064), Race17 (strain P7074), and Race19 (strain P7076) were 30.87, 28.22, and 23.33%, respectively. On the medium containing 2.5 mM LA, the bacteriostatic rates for Race2, Race 7, Race17, and Race19 were 64.84, 57.06, 42.16, and 62.86%, respectively. Higher concentrations of LA exacerbated its toxicity to various *P. sojae* races.

### Lauric Acid Derivatives Had Lower Toxicity to *P. sojae*

**FIGURE 2:**
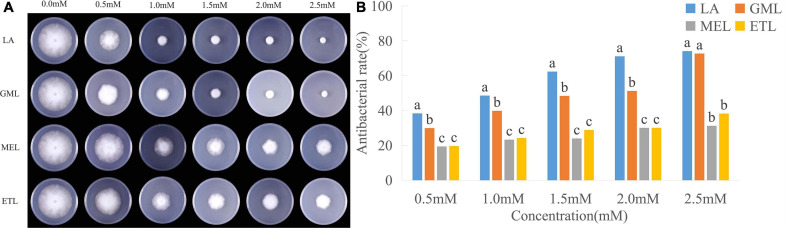
Inhibitory effects of lauric acid (LA) and its derivatives to *P. sojae*. **(A)** The mycelia of *P. sojae* (Race2) were cultured on media containing LA, glycerol monolaurate (GML), methyl laurate (MEL), or ethyl laurate (ETL) for 5 days. **(B)** The diameter of each colony was measured and statistical analyzed via software Statistical Product and Service Solutions (SPSS). Means with different letters are significantly different (*p* < 0.05).

Fatty acids can merge into cell membranes, and their hydrophilicity and lipophilicity likely affect their antimicrobial activity. To verify this hypothesis, we analyzed the mycelial growth diameter of *P. sojae* (Race2) grown under different concentrations of LA and its derivatives, GML, MEL, and ETL. The diameters and bacteriostatic rates of mycelia treated with GML, MEL, and ETL at 0.5 mM were 50.80, 57.53, and 57.37 mm, and 27.32, 17.70, and 17.93%, respectively. For LA, the corresponding values were 45.40 mm and 35.05%, indicating that its toxicity is higher than that of the three derivatives tested (Figure 2 and Supplementary Table 2). The inhibitory effect of LA of *P. sojae* was the most significant at the lowest concentration (0.5 mM). In summary, the toxicity effects to *P. sojae* were the highest for LA, followed by GML, ETL, and MEL.

### Lauric Acid Effects on the Development of *P. sojae* Mycelia and Sporangium

**FIGURE 3:**
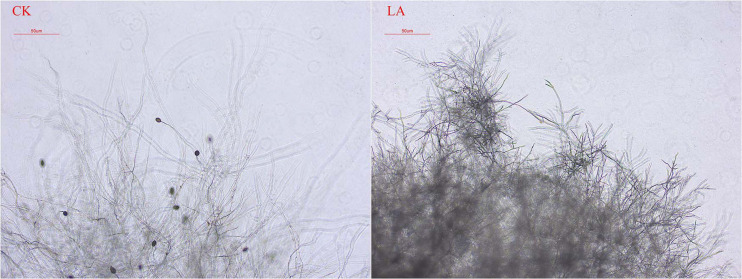
Effect of LA on *P. sojae* mycelial morphology. Mycelia were treated with LA or the same volume of water (CK) for 2 h before their morphology was analyzed. Mycelial growth after LA treatment was disorderly, the terminal branch number increased significantly, and the formation of zoosporangium was inhibited. In CK, the mycelium grew normally and expanded without any distortion, bending, or increased terminal branching. The experiment was repeated three times with similar results.

We observed the morphology of *P. sojae* hyphae with an optical microscope. LA treatments led to hyphae with more branches, distortions, bending, and nodules than those in the control group. Moreover, LA-treated hyphae had few zoosporangia (Figure 3).

To explore how LA and its derivatives affect *P. sojae* zoosporangium formation and zoospore release, we determined the number of zoosporangia formed and calculated the inhibition effect of each chemical. The number of zoosporangia was reduced with 0.16 mM LA or GML when compared with the water control; no zoosporangia were observed with 0.64 mM treatments. With 1.28 mM MEL or ETL, a small number of zoosporangia were obtained (Supplementary Table 3).

Zoosporangia formed in the water could not release zoospores when the LA or GML concentration increased to 0.64 mM. Interestingly, almost no zoospores were released from zoosporangia treated with 0.32 mM GML, which was significantly lower than what we observed for LA, MEL, or ETL (Supplementary Table 3).

### Lauric Acid and Its Derivatives Affect the Germination of *P. sojae* Zoospores

**FIGURE 4:**
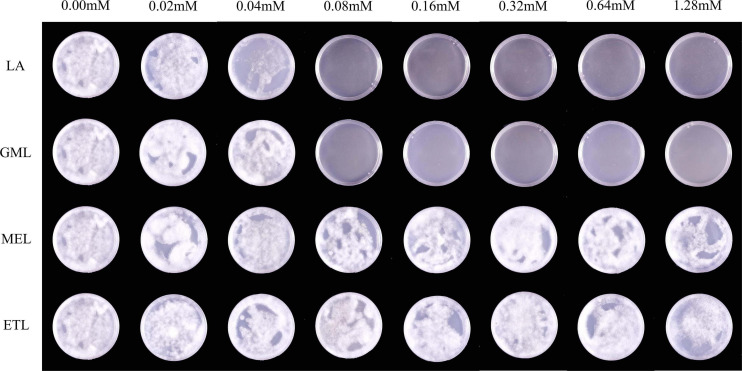
The effects of LA and its derivatives on *P. sojae* zoospore germination. To observe zoospore germination, zoospore suspensions were smeared on V8 medium containing LA, GML, MEL, or ETL at different concentrations. The experiment was repeated three times with similar results. Means with different letters are significantly different (Statistical Product and Service Solutions (SPSS), *p* < 0.05).

To explore the influence of LA and its derivatives on *P. sojae* zoospore germination, we evenly daubed zoospore suspensions onto media containing various concentrations of LA, GML, MEL, or ETL. Compared to the V8 medium control, LA and GML at 0.08 mM significantly inhibited zoospore germination, and few zoospores could form individual colonies (Figure 4). However, MEL and ETL had weak inhibitory effects on zoospore germination at the maximum concentration of 1.28 mM (Figure 4 and Supplementary Table 4).

### Lauric Acid Induced Cell Membrane Damage and Cell Substance Leakage in *P. sojae*

**FIGURE 5:**
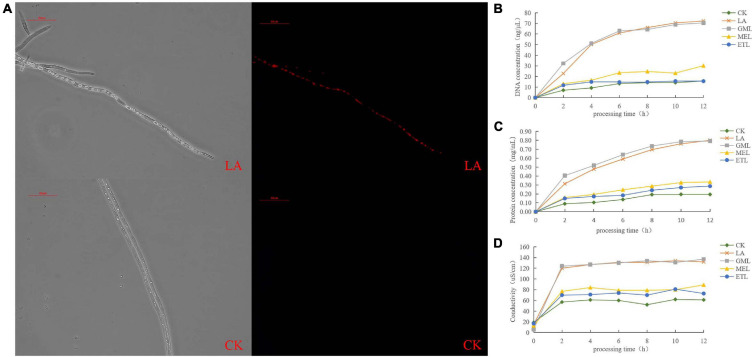
Effect of LA on *P. sojae* cell membrane integrity. **(A)** *P. sojae* hyphae nuclei stained with propidium iodide (PI) after exposure to LA (upper image) or the same amount of water (lower image) and observed under a microscope. **(B)** Effects of LA and its derivatives, GML, MEL, or ETL, on DNA content. **(C)** Effects of LA GML, MEL, and ETL on protein content. **(D)** Effects of LA, GML, MEL, and ETL on solution conductivity. Means with different letters are significantly different (SPSS, *p* < 0.05).

Propidium iodate is a DNA-binding and a cell-membrane–impermeable dye, usually used as a marker for membrane integrity and cell viability; PI cannot cross the membrane of live cells but can penetrate and stain the cell nucleus red if the cell membrane is damaged. We PI-stained *P. sojae* hyphae nuclei after exposure to LA and observed that the cell membrane was severely damaged, resulting in red fluorescence in the nucleus (Figure 5A).

To further investigate the potential damage to the cell membrane, we analyzed cell substance leakage in *P. sojae* mycelia treated with LA and its derivatives, GML, MEL, and ETL. Total DNA and protein concentrations were monitored every 2 h in the growth media. DNA and protein contents increased significantly over time and gradually flattened out in LA and GML solutions but changed only slightly in CK, MEL, or ETL solutions (Figures 5B, C). The conductibility of the five tested solutions increased in the first 2 h, but it was significantly higher in LA and GML than in CK, MEL, or ETL (Figure 5D). The results indicated that the *P. sojae* DNA and proteins penetrated the solution upon treatment with LA or GML, probably because the cell membrane was damaged or destroyed.

### Host Security of Treatments With LA and Its Derivatives on Soybean Plants

To understand the security of LA and its derivatives on host plants, we applied different concentrations of LA, GML, MEL, or ETL in the pot-planting holes; soybean seedling height was measured at 7 and 14 days after planting. When LA and GML concentrations were lower than 0.3 g a.i./plant, the treatments promoted soybean growth, whereas the application of 0.3 g a.i./plant did not influence plant growth, and there was no significant difference in height compared with the control group. At 0.6 g a.i./plant, MEL and ETL treatments did not significantly affect plant growth compared to the control at the two time points. However, concentrations higher than 0.6 g a.i./plant were harmful to plants and inhibited their growth (Supplementary Table 5).

### Potential of LA and Its Derivatives as Biological Control Agents

**FIGURE 6:**
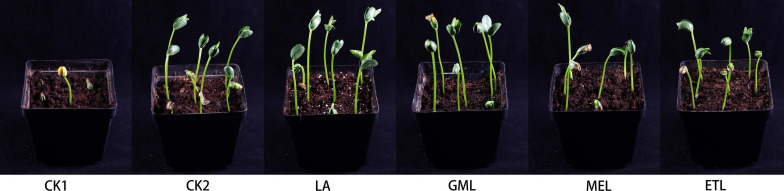
Potted experiment of *P. sojae* control by LA and its derivatives. The soil was treated with LA, GML, MEL, or ETL. In CK1, no agent added to the soil. In CK2, no *P. sojae* mycelium was added to the soil. Plant height was measured 5 days after planting. The experiment was repeated three times with similar results. Means with different letters are significantly different (SPSS, *p* < 0.05).

The mycelium and oospores of *P. sojae* formed on V8 medium were homogenized using a blender and mixed with potted soil before LA, and its derivatives, GML, MEL, or ETL, were added to the pots. Seeds of the *P. sojae*–susceptible soybean cultivar Williams (lacking *Rps* resistance genes) were sowed in the processed soil, and soybean height was measured after 5 days. Potted soybean plants grown in soil treated with LA or its derivatives had similar growth patterns than those in sterilized soil (CK2). However, most seedlings were killed by *P. sojae* in inoculated soil when plants were planted without LA, GML, MEL, or ETL treatments (CK1) (Figure 6).

## Discussion

Because of the expanding global population and the increasingly stringent environmental, toxicological, and regulatory requirements, plant pathogen control remains a constant need, and the products suitable for phytopathogen management are further limited. NPs are primary or secondary metabolites produced by living cells and have been an important source and template for the development of novel environmental-friendly agrochemicals. The metabolites synthesized in response to pathogen infection, such as leaf VOCs, have been particularly important and have functional benefits in multiple aspects of plant defense. Several VOCs are produced and emitted rapidly when plants respond to biotic stress, and such compounds require further exploration as leads for novel crop protection chemistry. VOCs tend to be chemically complex, and few are used directly for agricultural pathogen control. Nevertheless, it is technically possible to identify the effective constituents in VOCs and modify them as commercial products, transforming many NPs into agriculturally suitable molecules for pathogen control.

Here, we identified the components of soybean leaf volatiles produced in incompatible interactions and compatible interactions with *P. sojae* and found that LA was emitted specificity in a resistant cultivar. As previously reported, LA inhibits the mycelial growth of phytopathogenic fungi. Additionally, LA significantly reduces the *R. solani* and *P. ultimum* mycelial growth in agar culture at the concentration of 100 μm or greater, whereas no fungal growth occurred in liquid culture at concentrations greater than 50 μm (Walters et al., 2003). In this study, *P. sojae* mycelia growth was suppressed by LA, and the formation of zoosporangium was inhibited. This study provides the first report of the activity of LA against a *Phytophthora* pathogen and indicates the need for further research of its mechanism of action.

Lauric acid-treated mycelia grew disorderly, and the number of terminal branches increased significantly. Furthermore, the plasma membrane of *P. sojae* was disrupted or disintegrated by LA. MCFAs and monoglycerides are single-chain lipid amphiphiles that interact with phospholipid membranes as part of various biological activities. For example, they can have membrane-disruptive behavior against microbial pathogens on the human skin surface (Valle-González et al., 2018). LA has a minimum bactericidal concentration to *E. coli* at 1 mM (Kim and Rhee, 2013). Here, we found that LA can inhibit *P. sojae* growth at 0.5 mM, which means it has a greater effect on *Phytophthora* than on *E. coli*. Additionally, the concentration of cell substances, including DNA and protein, in water culture increased after LA treatment, and PI staining of the nucleus indicated that the *P. sojae* cell membrane was damaged.

It has been proposed that the hydrophilic and lipophilic characteristics of fatty acid derivatives affect their antibacterial activities according to their ability to incorporate into the bacterial cell membrane (Park et al., 2018; Valle-González et al., 2018). To verify this hypothesis, we selected three kinds of LA derivatives esterified with different non–fatty acid moieties and investigated whether the antibacterial activity from their precursor (i.e., LA) was retained or lost. GML had comparative bacteriostatic and bactericidal effects against *P. sojae* to LA, whereas MEL and ETL had weaker inhibitory activity. The antimicrobial properties of LA, GML, and their ester derivatives may be attributed to physicochemical processes and their interference with various cellular processes. GML is 200 times more effective than LA in bactericidal activity (Schlievert and Peterson, 2012), but, in this study, LA had similar effects to *P. sojae*, which suggests differences in the inhibitory mechanisms involved.

Glycerol monolaurate has a potent antimicrobial activity, and LA has the ability to convert into GML, which can destroy the lipid membrane of bacteria (Jin et al., 2021). The high levels of activity of capric and lauric acids, and particularly that of monocaprin, are notable and suggest that these lipids have specific antichlamydial activities (Bergsson et al., 1998). A recent study demonstrated that the potencies of saturated FAs increased sharply by lowering the pH, and a decrease of only 0.5 pH units could cause a change from non-lethal to lethal conditions. Conversely, the bactericidal action of GLM was not pH-dependent (Sun et al., 2003). That means GML is more environmentally stable and likely more suitable for crop protection.

As the main component of coconut oil and breast milk, LA is usually part of regular diets and feed additives, showing potent antimicrobial effects and lack of toxicity (Zeiger et al., 2017; Khan et al., 2020; Zhang et al., 2020). In the host security test, with concentrations lower than 0.3 g a.i./plant, both LA and GML could promote soybean growth; however, higher concentrations may negatively influence host growth.

None of the natural and eco-friendly chemical alternatives currently registered and available have the full spectrum of activity and versatility of methyl bromide as preplant soil fumigants (Duniway, 2002). Based on the results described here, LA and GML have potent antimicrobial effects and positive effects regulating plant growth. Hence, they may be promising substitutes for traditional anti-*Phytophthora* agents.

## Data Availability Statement

The original contributions presented in the study are included in the article/Supplementary Material, further inquiries can be directed to the corresponding authors.

## Author Contributions

QW and QX designed the experiments. CH, TG, and WG wrote the manuscript, performed the experiments, and analyzed the data. YW performed the GC–MS experiments and analyzed the data. QX, JW, and XT participated in manuscript revision or experiments. QW revised the manuscript and provided the funding for this research. All authors contributed to the article and approved the submitted version.

## Conflict of Interest

YW was employed by company Shimadzu (China) Co., Ltd. The remaining authors declare that the research was conducted in the absence of any commercial or financial relationships that could be construed as a potential conflict of interest.

## Publisher’s Note

All claims expressed in this article are solely those of the authors and do not necessarily represent those of their affiliated organizations, or those of the publisher, the editors and the reviewers. Any product that may be evaluated in this article, or claim that may be made by its manufacturer, is not guaranteed or endorsed by the publisher.
