# Supplementary material for: Lauric Acid Is a Potent Biological Control Agent That Damages the Cell Membrane of Phytophthora sojae
Source: Front Microbiol. 2021 Aug 5;12:666761. doi: 10.3389/fmicb.2021.666761 (PMC8374439; doi:10.3389/fmicb.2021.666761)
Supplement: Supplementary file 1 [file Presentation_1.PPTX]

## Slide 1
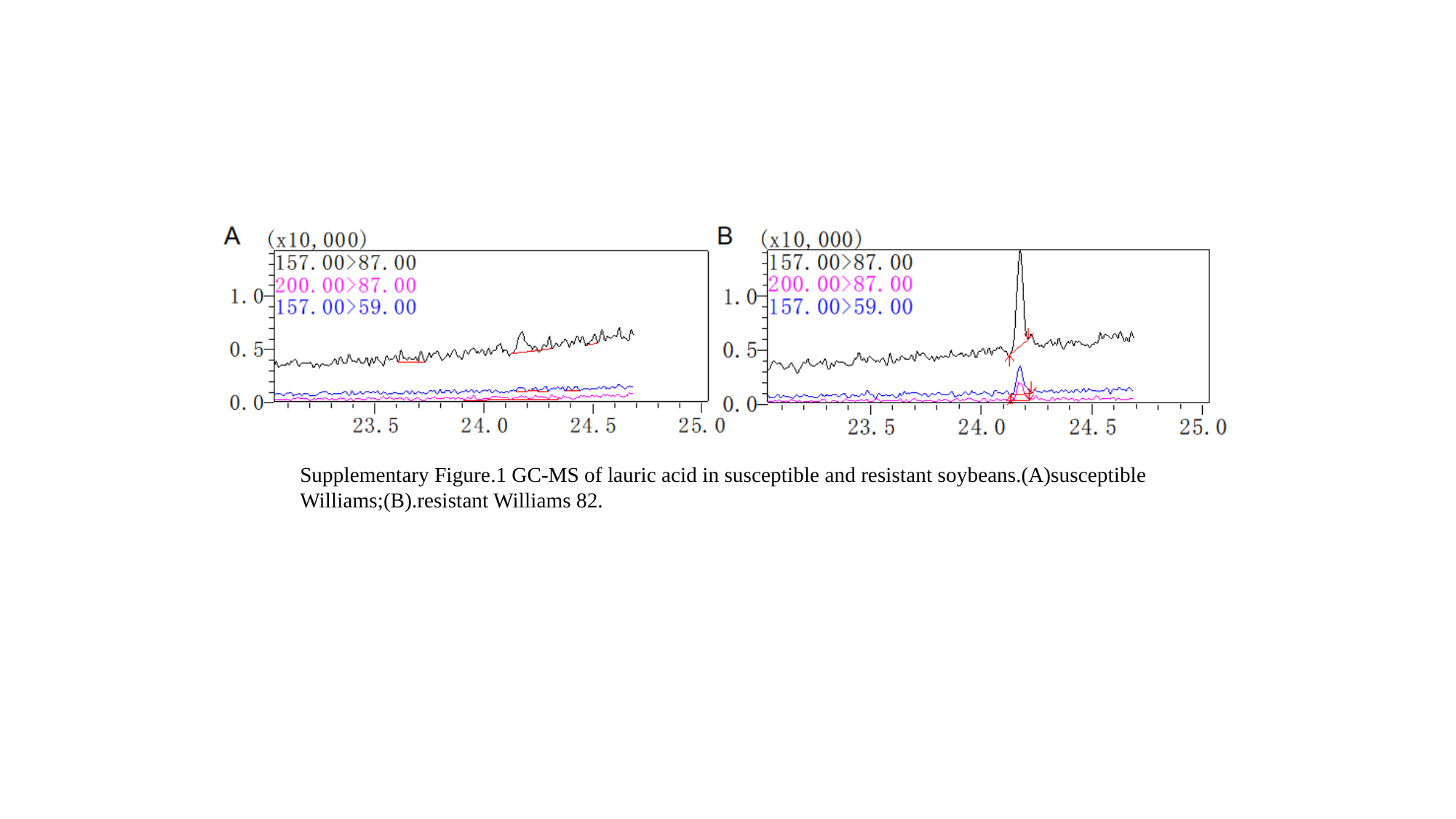

Supplementary Figure.1 GC-MS of lauric acid in susceptible and resistant soybeans.(A)susceptible Williams;(B).resistant Williams 82.

## Slide 2
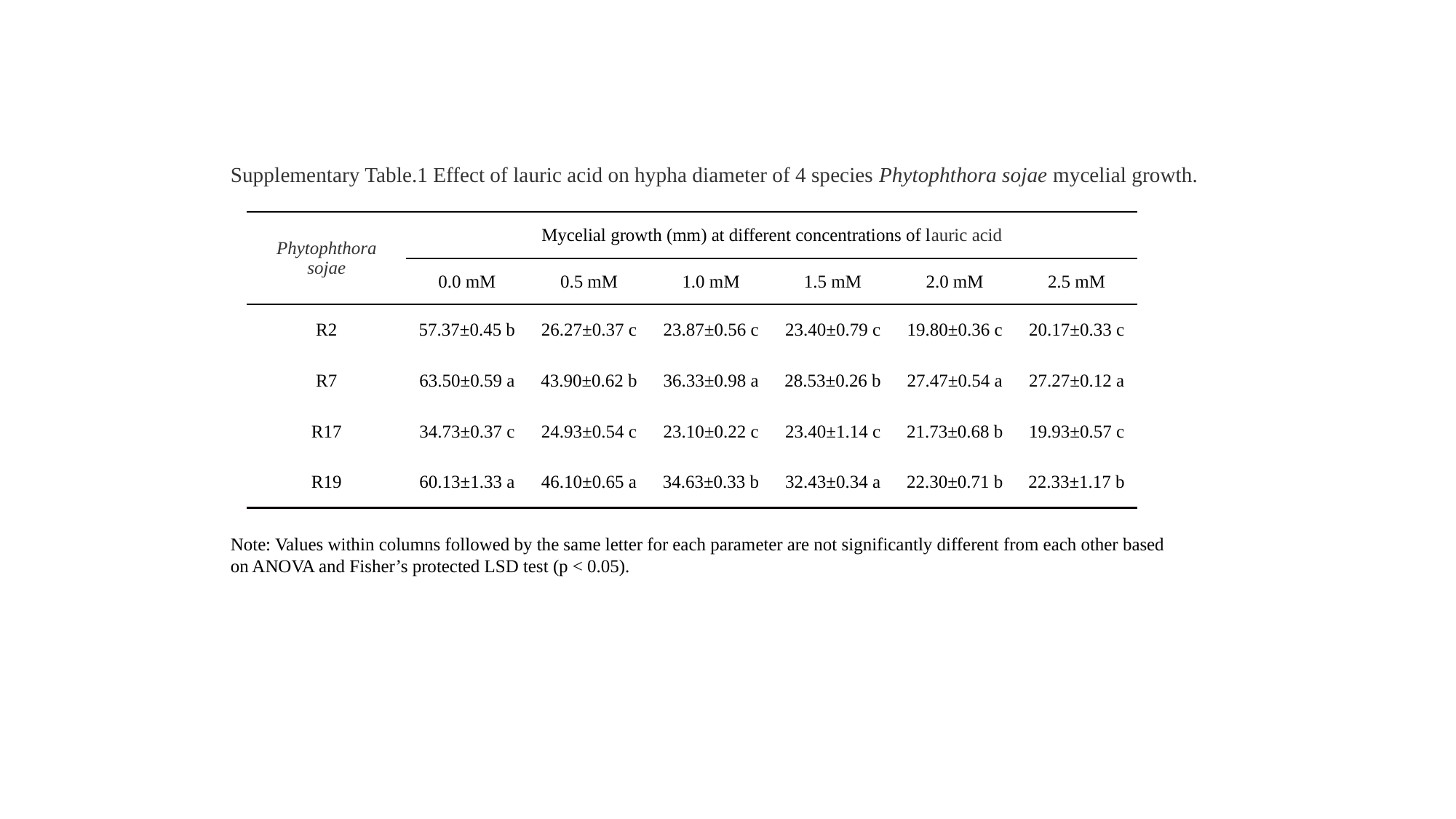

Supplementary Table.1 Effect of lauric acid on hypha diameter of 4 species Phytophthora sojae mycelial growth.
| Phytophthora sojae | Mycelial growth (mm) at different concentrations of lauric acid | | | | | |
| --- | --- | --- | --- | --- | --- | --- |
| | 0.0 mM | 0.5 mM | 1.0 mM | 1.5 mM | 2.0 mM | 2.5 mM |
| R2 | 57.37±0.45 b | 26.27±0.37 c | 23.87±0.56 c | 23.40±0.79 c | 19.80±0.36 c | 20.17±0.33 c |
| R7 | 63.50±0.59 a | 43.90±0.62 b | 36.33±0.98 a | 28.53±0.26 b | 27.47±0.54 a | 27.27±0.12 a |
| R17 | 34.73±0.37 c | 24.93±0.54 c | 23.10±0.22 c | 23.40±1.14 c | 21.73±0.68 b | 19.93±0.57 c |
| R19 | 60.13±1.33 a | 46.10±0.65 a | 34.63±0.33 b | 32.43±0.34 a | 22.30±0.71 b | 22.33±1.17 b |
Note: Values within columns followed by the same letter for each parameter are not significantly different from each other based on ANOVA and Fisher’s protected LSD test (p < 0.05).

## Slide 3
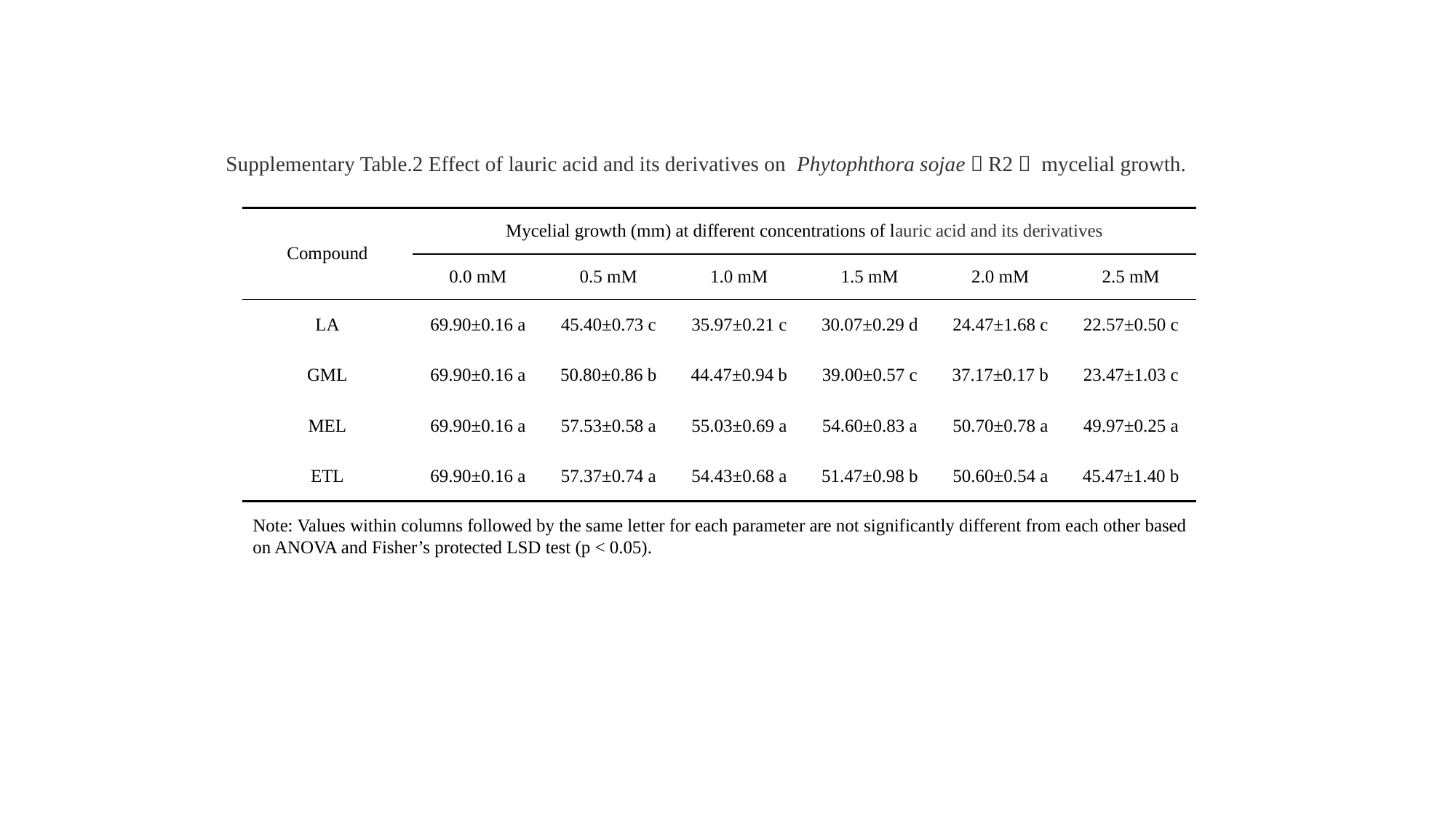

Supplementary Table.2 Effect of lauric acid and its derivatives on Phytophthora sojae（R2） mycelial growth.
| Compound | Mycelial growth (mm) at different concentrations of lauric acid and its derivatives | | | | | |
| --- | --- | --- | --- | --- | --- | --- |
| | 0.0 mM | 0.5 mM | 1.0 mM | 1.5 mM | 2.0 mM | 2.5 mM |
| LA | 69.90±0.16 a | 45.40±0.73 c | 35.97±0.21 c | 30.07±0.29 d | 24.47±1.68 c | 22.57±0.50 c |
| GML | 69.90±0.16 a | 50.80±0.86 b | 44.47±0.94 b | 39.00±0.57 c | 37.17±0.17 b | 23.47±1.03 c |
| MEL | 69.90±0.16 a | 57.53±0.58 a | 55.03±0.69 a | 54.60±0.83 a | 50.70±0.78 a | 49.97±0.25 a |
| ETL | 69.90±0.16 a | 57.37±0.74 a | 54.43±0.68 a | 51.47±0.98 b | 50.60±0.54 a | 45.47±1.40 b |
Note: Values within columns followed by the same letter for each parameter are not significantly different from each other based on ANOVA and Fisher’s protected LSD test (p < 0.05).

## Slide 4
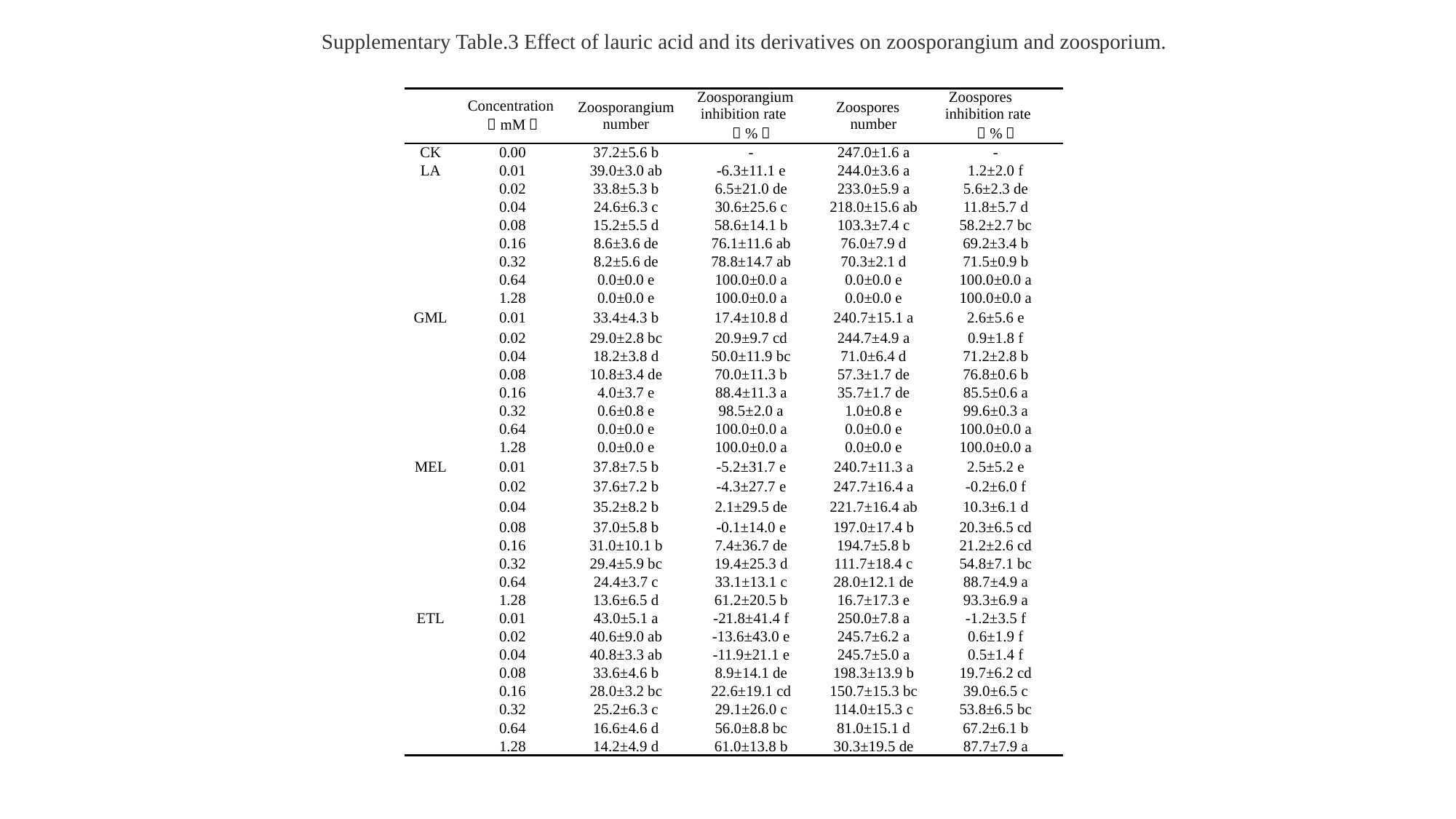

Supplementary Table.3 Effect of lauric acid and its derivatives on zoosporangium and zoosporium.
| | Concentration（mM） | Zoosporangium number | Zoosporangium inhibition rate （%） | Zoospores number | Zoospores inhibition rate （%） |
| --- | --- | --- | --- | --- | --- |
| CK | 0.00 | 37.2±5.6 b | - | 247.0±1.6 a | - |
| LA | 0.01 | 39.0±3.0 ab | -6.3±11.1 e | 244.0±3.6 a | 1.2±2.0 f |
| | 0.02 | 33.8±5.3 b | 6.5±21.0 de | 233.0±5.9 a | 5.6±2.3 de |
| | 0.04 | 24.6±6.3 c | 30.6±25.6 c | 218.0±15.6 ab | 11.8±5.7 d |
| | 0.08 | 15.2±5.5 d | 58.6±14.1 b | 103.3±7.4 c | 58.2±2.7 bc |
| | 0.16 | 8.6±3.6 de | 76.1±11.6 ab | 76.0±7.9 d | 69.2±3.4 b |
| | 0.32 | 8.2±5.6 de | 78.8±14.7 ab | 70.3±2.1 d | 71.5±0.9 b |
| | 0.64 | 0.0±0.0 e | 100.0±0.0 a | 0.0±0.0 e | 100.0±0.0 a |
| | 1.28 | 0.0±0.0 e | 100.0±0.0 a | 0.0±0.0 e | 100.0±0.0 a |
| GML | 0.01 | 33.4±4.3 b | 17.4±10.8 d | 240.7±15.1 a | 2.6±5.6 e |
| | 0.02 | 29.0±2.8 bc | 20.9±9.7 cd | 244.7±4.9 a | 0.9±1.8 f |
| | 0.04 | 18.2±3.8 d | 50.0±11.9 bc | 71.0±6.4 d | 71.2±2.8 b |
| | 0.08 | 10.8±3.4 de | 70.0±11.3 b | 57.3±1.7 de | 76.8±0.6 b |
| | 0.16 | 4.0±3.7 e | 88.4±11.3 a | 35.7±1.7 de | 85.5±0.6 a |
| | 0.32 | 0.6±0.8 e | 98.5±2.0 a | 1.0±0.8 e | 99.6±0.3 a |
| | 0.64 | 0.0±0.0 e | 100.0±0.0 a | 0.0±0.0 e | 100.0±0.0 a |
| | 1.28 | 0.0±0.0 e | 100.0±0.0 a | 0.0±0.0 e | 100.0±0.0 a |
| MEL | 0.01 | 37.8±7.5 b | -5.2±31.7 e | 240.7±11.3 a | 2.5±5.2 e |
| | 0.02 | 37.6±7.2 b | -4.3±27.7 e | 247.7±16.4 a | -0.2±6.0 f |
| | 0.04 | 35.2±8.2 b | 2.1±29.5 de | 221.7±16.4 ab | 10.3±6.1 d |
| | 0.08 | 37.0±5.8 b | -0.1±14.0 e | 197.0±17.4 b | 20.3±6.5 cd |
| | 0.16 | 31.0±10.1 b | 7.4±36.7 de | 194.7±5.8 b | 21.2±2.6 cd |
| | 0.32 | 29.4±5.9 bc | 19.4±25.3 d | 111.7±18.4 c | 54.8±7.1 bc |
| | 0.64 | 24.4±3.7 c | 33.1±13.1 c | 28.0±12.1 de | 88.7±4.9 a |
| | 1.28 | 13.6±6.5 d | 61.2±20.5 b | 16.7±17.3 e | 93.3±6.9 a |
| ETL | 0.01 | 43.0±5.1 a | -21.8±41.4 f | 250.0±7.8 a | -1.2±3.5 f |
| | 0.02 | 40.6±9.0 ab | -13.6±43.0 e | 245.7±6.2 a | 0.6±1.9 f |
| | 0.04 | 40.8±3.3 ab | -11.9±21.1 e | 245.7±5.0 a | 0.5±1.4 f |
| | 0.08 | 33.6±4.6 b | 8.9±14.1 de | 198.3±13.9 b | 19.7±6.2 cd |
| | 0.16 | 28.0±3.2 bc | 22.6±19.1 cd | 150.7±15.3 bc | 39.0±6.5 c |
| | 0.32 | 25.2±6.3 c | 29.1±26.0 c | 114.0±15.3 c | 53.8±6.5 bc |
| | 0.64 | 16.6±4.6 d | 56.0±8.8 bc | 81.0±15.1 d | 67.2±6.1 b |
| | 1.28 | 14.2±4.9 d | 61.0±13.8 b | 30.3±19.5 de | 87.7±7.9 a |

## Slide 5
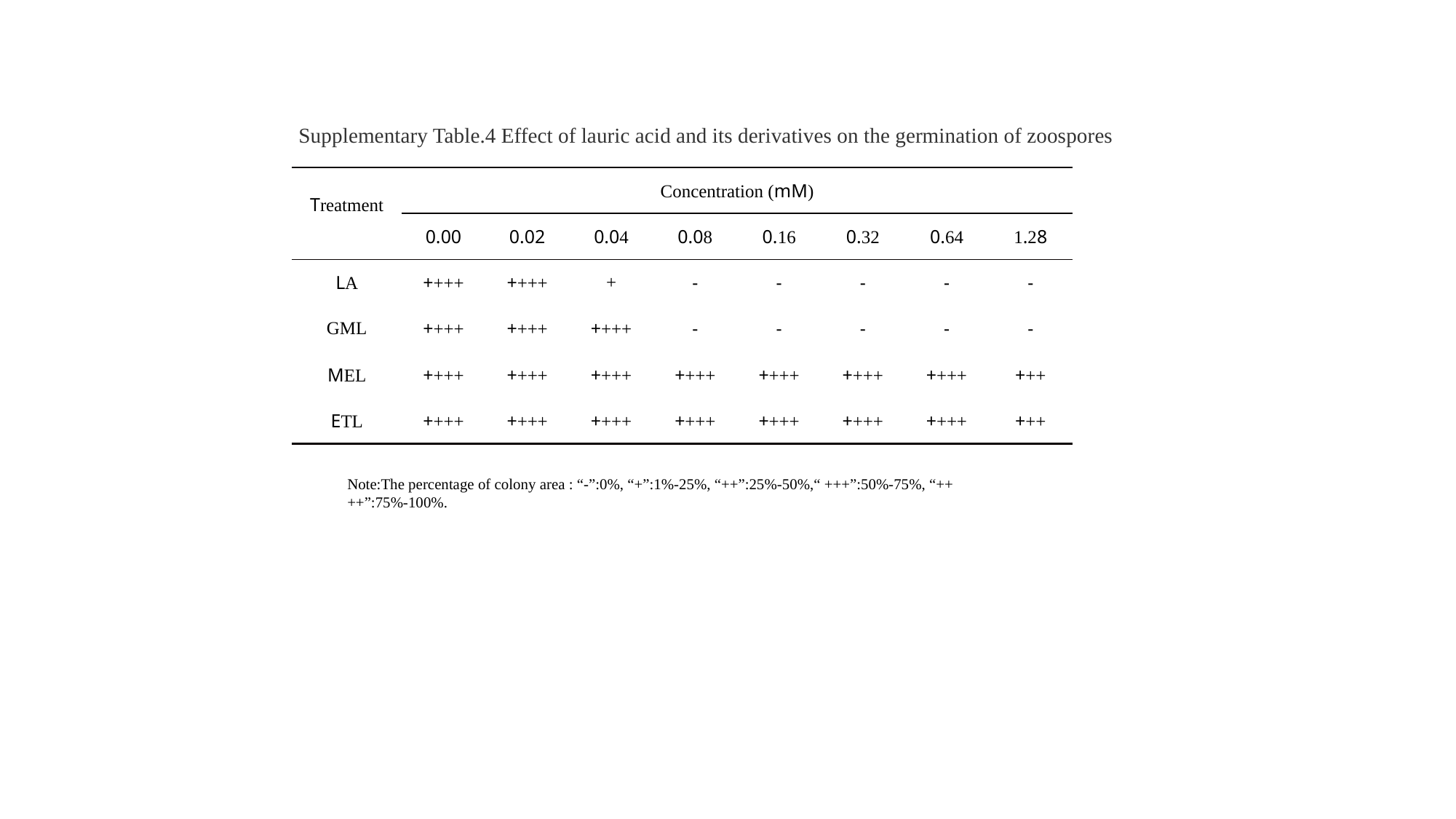

Supplementary Table.4 Effect of lauric acid and its derivatives on the germination of zoospores
| Treatment | Concentration (mM) | | | | | | | |
| --- | --- | --- | --- | --- | --- | --- | --- | --- |
| | 0.00 | 0.02 | 0.04 | 0.08 | 0.16 | 0.32 | 0.64 | 1.28 |
| LA | ++++ | ++++ | + | - | - | - | - | - |
| GML | ++++ | ++++ | ++++ | - | - | - | - | - |
| MEL | ++++ | ++++ | ++++ | ++++ | ++++ | ++++ | ++++ | +++ |
| ETL | ++++ | ++++ | ++++ | ++++ | ++++ | ++++ | ++++ | +++ |
Note:The percentage of colony area : “-”:0%, “+”:1%-25%, “++”:25%-50%,“ +++”:50%-75%, “++++”:75%-100%.

## Slide 6
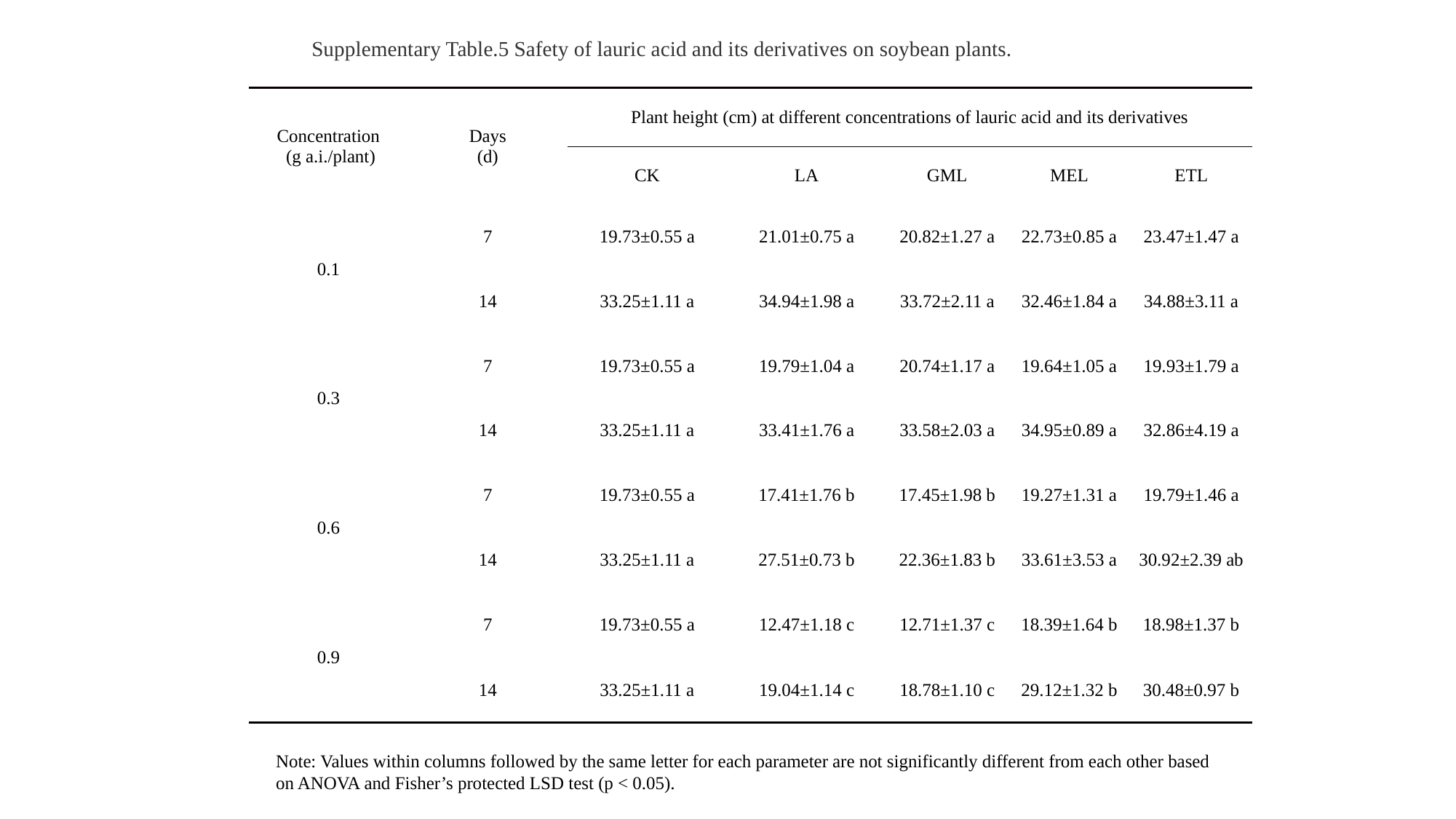

Supplementary Table.5 Safety of lauric acid and its derivatives on soybean plants.
| Concentration (g a.i./plant) | Days (d) | Plant height (cm) at different concentrations of lauric acid and its derivatives | | | | |
| --- | --- | --- | --- | --- | --- | --- |
| | | CK | LA | GML | MEL | ETL |
| 0.1 | 7 | 19.73±0.55 a | 21.01±0.75 a | 20.82±1.27 a | 22.73±0.85 a | 23.47±1.47 a |
| | 14 | 33.25±1.11 a | 34.94±1.98 a | 33.72±2.11 a | 32.46±1.84 a | 34.88±3.11 a |
| 0.3 | 7 | 19.73±0.55 a | 19.79±1.04 a | 20.74±1.17 a | 19.64±1.05 a | 19.93±1.79 a |
| | 14 | 33.25±1.11 a | 33.41±1.76 a | 33.58±2.03 a | 34.95±0.89 a | 32.86±4.19 a |
| 0.6 | 7 | 19.73±0.55 a | 17.41±1.76 b | 17.45±1.98 b | 19.27±1.31 a | 19.79±1.46 a |
| | 14 | 33.25±1.11 a | 27.51±0.73 b | 22.36±1.83 b | 33.61±3.53 a | 30.92±2.39 ab |
| 0.9 | 7 | 19.73±0.55 a | 12.47±1.18 c | 12.71±1.37 c | 18.39±1.64 b | 18.98±1.37 b |
| | 14 | 33.25±1.11 a | 19.04±1.14 c | 18.78±1.10 c | 29.12±1.32 b | 30.48±0.97 b |
Note: Values within columns followed by the same letter for each parameter are not significantly different from each other based on ANOVA and Fisher’s protected LSD test (p < 0.05).

## Slide 7
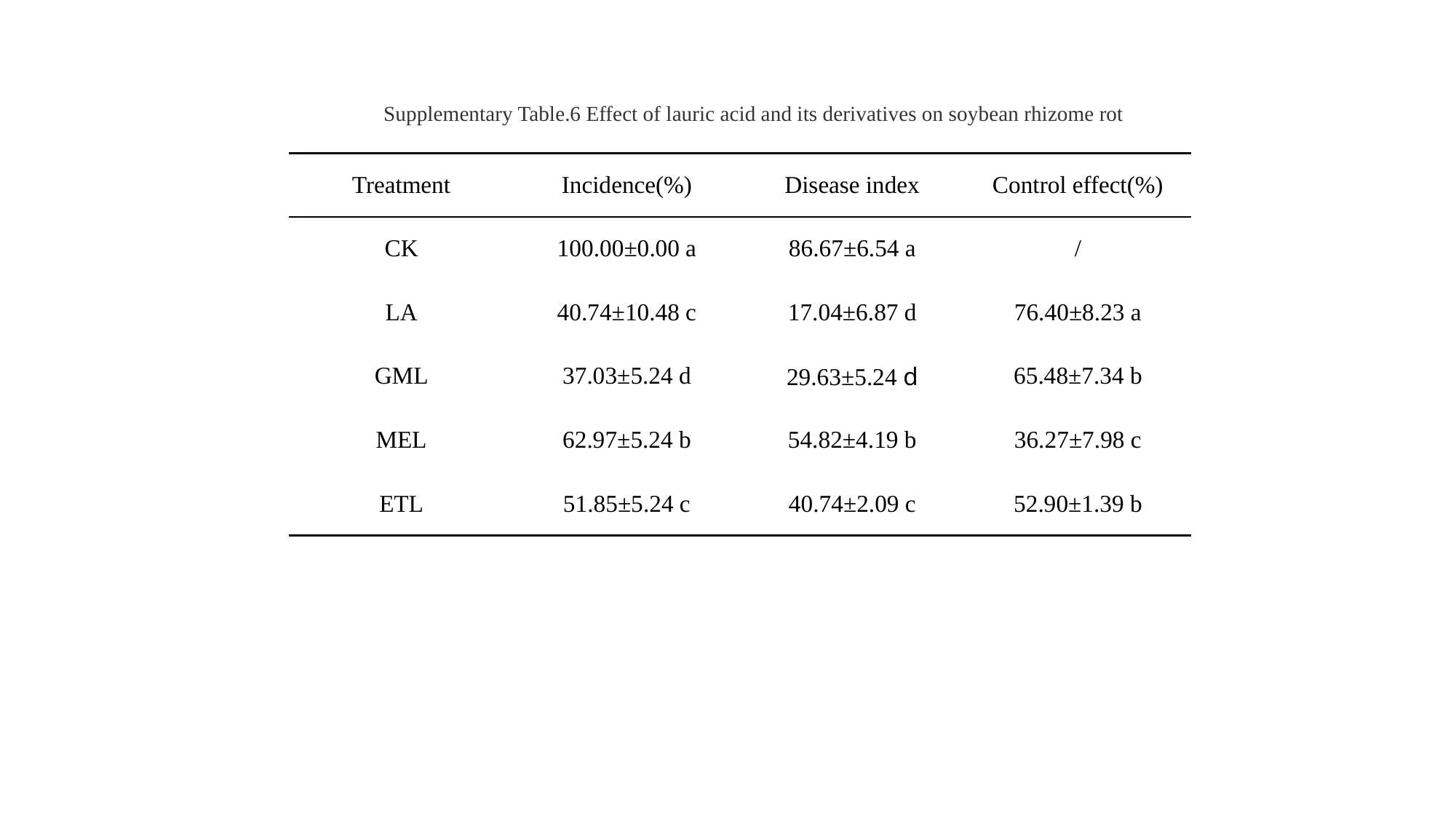

Supplementary Table.6 Effect of lauric acid and its derivatives on soybean rhizome rot
| Treatment | Incidence(%) | Disease index | Control effect(%) |
| --- | --- | --- | --- |
| CK | 100.00±0.00 a | 86.67±6.54 a | / |
| LA | 40.74±10.48 c | 17.04±6.87 d | 76.40±8.23 a |
| GML | 37.03±5.24 d | 29.63±5.24 d | 65.48±7.34 b |
| MEL | 62.97±5.24 b | 54.82±4.19 b | 36.27±7.98 c |
| ETL | 51.85±5.24 c | 40.74±2.09 c | 52.90±1.39 b |
